# Supplementary material for: Litsea glaucescens Kuth possesses bactericidal activity against Listeria monocytogenes
Source: PeerJ. 2023 Dec 1;11:e16522. doi: 10.7717/peerj.16522 (PMC10695109; doi:10.7717/peerj.16522)
Supplement: Supplemental Information 2 — The figures show all one and two dimension spectra obtained for Pinocembrin. [file peerj-11-16522-s002.docx]

***Litsea glaucescens* Kuth possesses bactericidal activity against *Listeria monocytogenes*.**

Carlos David Gress-Antonio^1^, Nallely Rivero-Perez^1^, Silvia Marquina Bahena^2^, Laura Alvarez^2^, Adrian Zaragoza-Bastida^1^, Víctor Manuel Martínez-Juarez^1^, Carolina G. Sosa-Gutiérrez^1^, Juan Ocampo-López^1^, Armando Zepeda-Bastida^1^, Deyanira Ojeda-Ramírez^1^

^1^ Área Académica de Medicina Veterinaria y Zootecnia, Universidad Autónoma del Estado de Hidalgo, Tulancingo de Bravo, Hidalgo,

^2^ Centro de Investigaciones Químicas, Universidad Autónoma del Estado de Morelos, Cuernavaca, Morelos, México

Corresponding Author:

Deyanira Ojeda-Ramírez^1^

Avenida Universidad Km1, Ex-hacienda de Aquetzalpa, Tulancingo. Hidalgo, 43600, Mexico.

Email address: [dojeda@uaeh.edu.mx](mailto:dojeda@uaeh.edu.mx)

Content

Figure S1. **.**^1^H NMR (500 MHz, DMSO-d_6_) of 5,7-dihydroxy flavanone (Pinocembrin, **1**).

Figure S2. **.**^1^H NMR (500 MHz, DMSO-d_6_ + D_2_O) of 5,7-dihydroxy flavanone (Pinocembrin, **1**).

Figure S3. **.**^1^H NMR (500 MHz, CDCl_3_-CD_3_OD) of 5,7-dihydroxy flavanone (Pinocembrin, **1**).

Figure S4. **.**^13^C NMR (125 MHz, CDCl_3_-CD_3_OD) of 5,7-dihydroxy flavanone (Pinocembrin, **1**).

Figure S5. COSY (500 MHz, CDCl_3_-CD_3_OD) of 5,7-dihydroxy flavanone (Pinocembrin, **1**).

Figure S6. HSQC (500 MHz, CDCl_3_-CD_3_OD) of 5,7-dihydroxy flavanone (Pinocembrin, **1**).


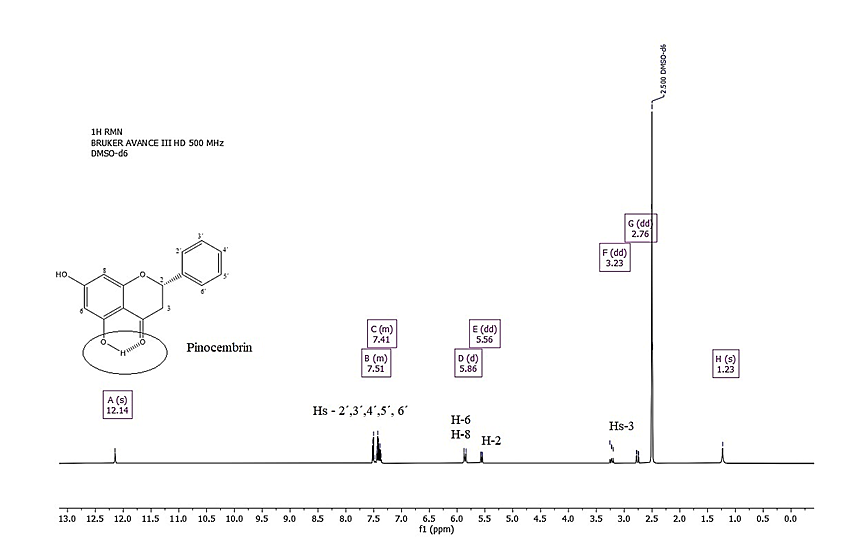


Figure S1. **.**^1^H NMR (500 MHz, DMSO-d_6_) of 5,7-dihydroxy flavanone (Pinocembrin, **1**).


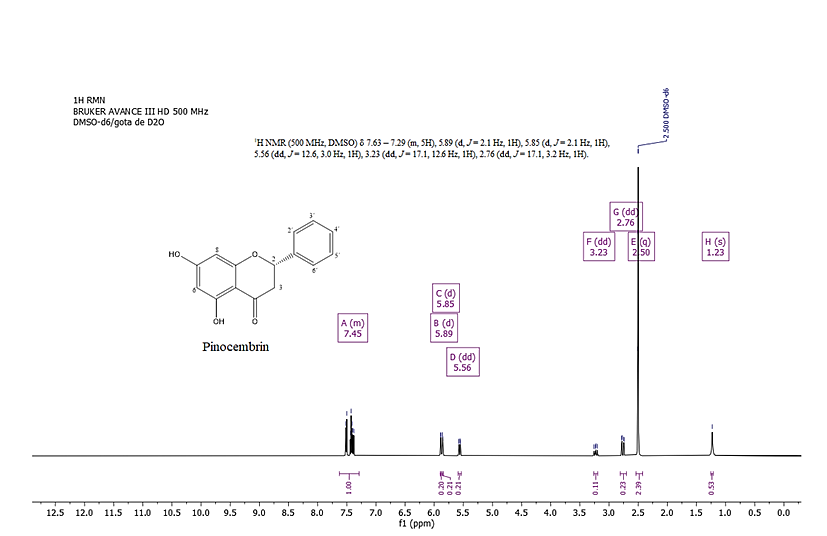


Figure S2. **.**^1^H NMR (500 MHz, DMSO-d_6_ + D_2_O) of 5,7-dihydroxy flavanone (Pinocembrin, **1**).


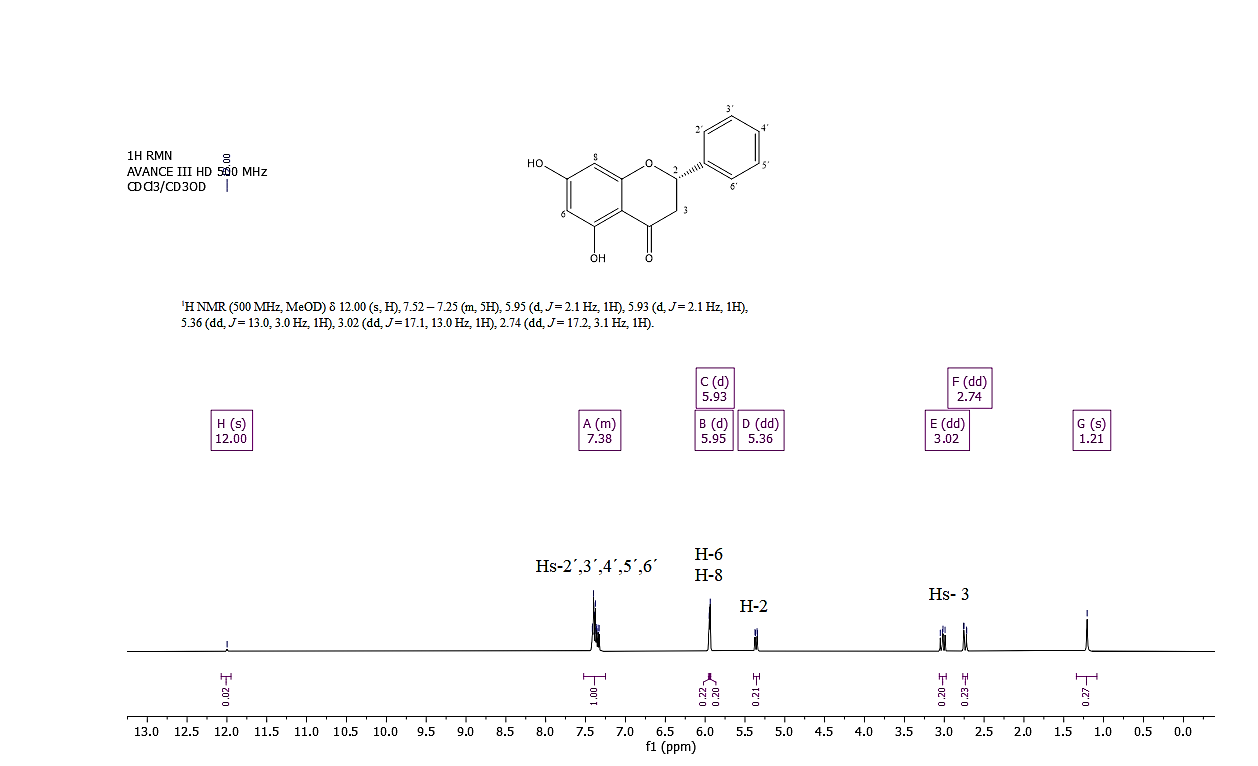


Figure S3. **.**^1^H NMR (500 MHz, CDCl_3_-CD_3_OD) of 5,7-dihydroxy flavanone (Pinocembrin, **1**).


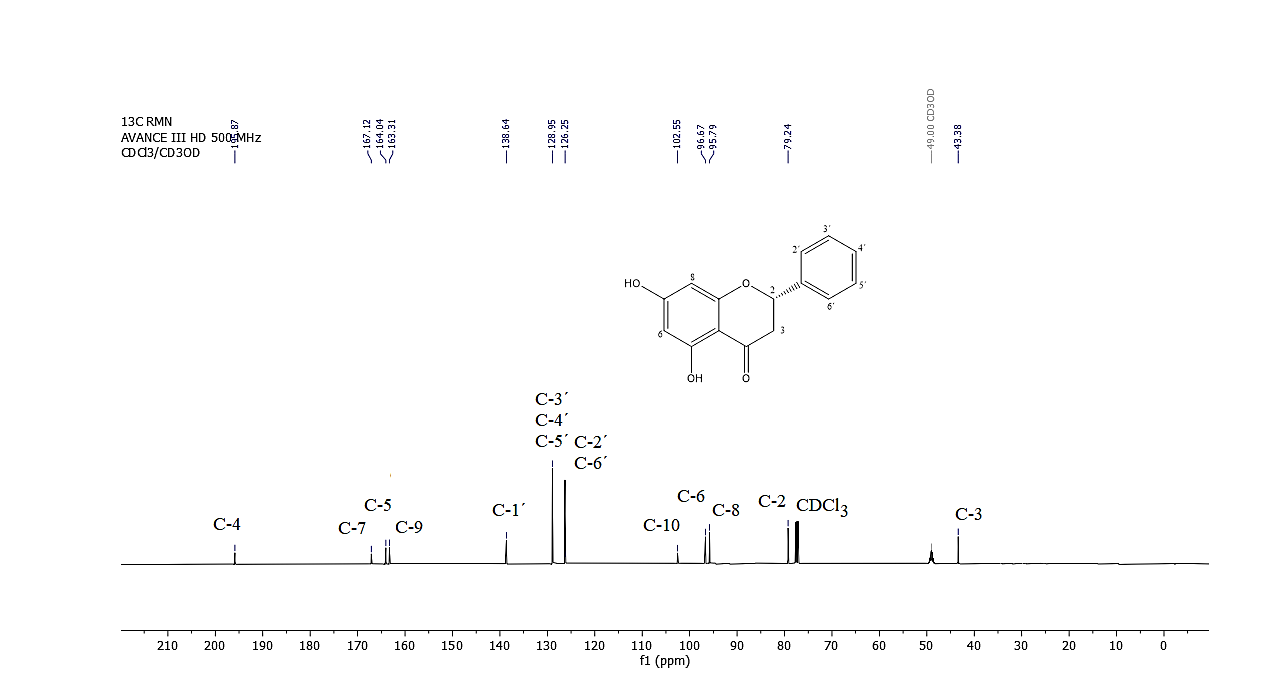


Figure S4. **.**^13^C NMR (125 MHz, CDCl_3_-CD_3_OD) of 5,7-dihydroxy flavanone (Pinocembrin, **1**).


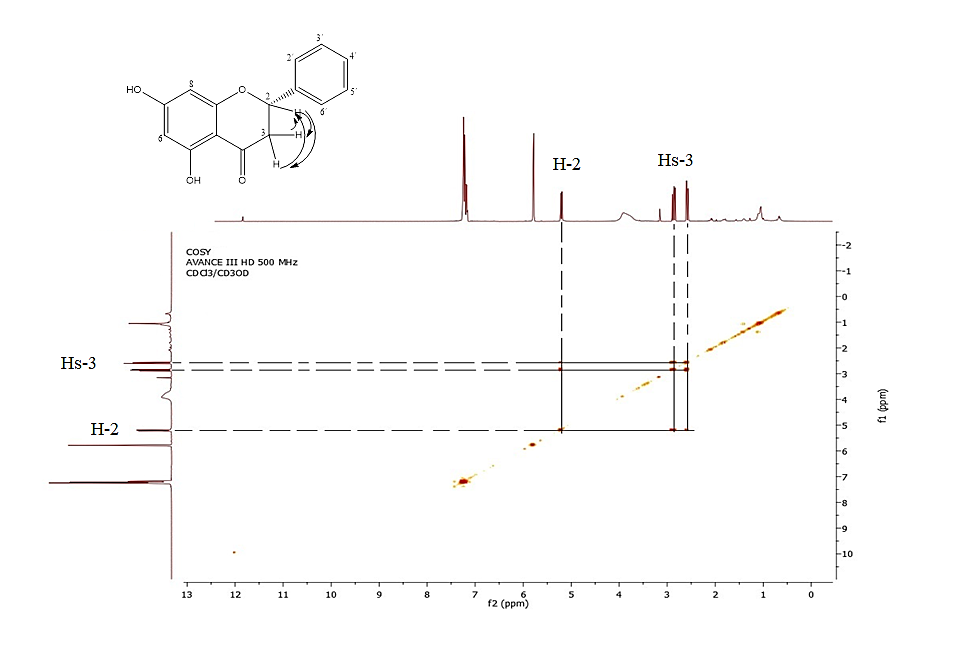


Figure S5. COSY (500 MHz, CDCl_3_-CD_3_OD) of 5,7-dihydroxy flavanone (Pinocembrin, **1**).


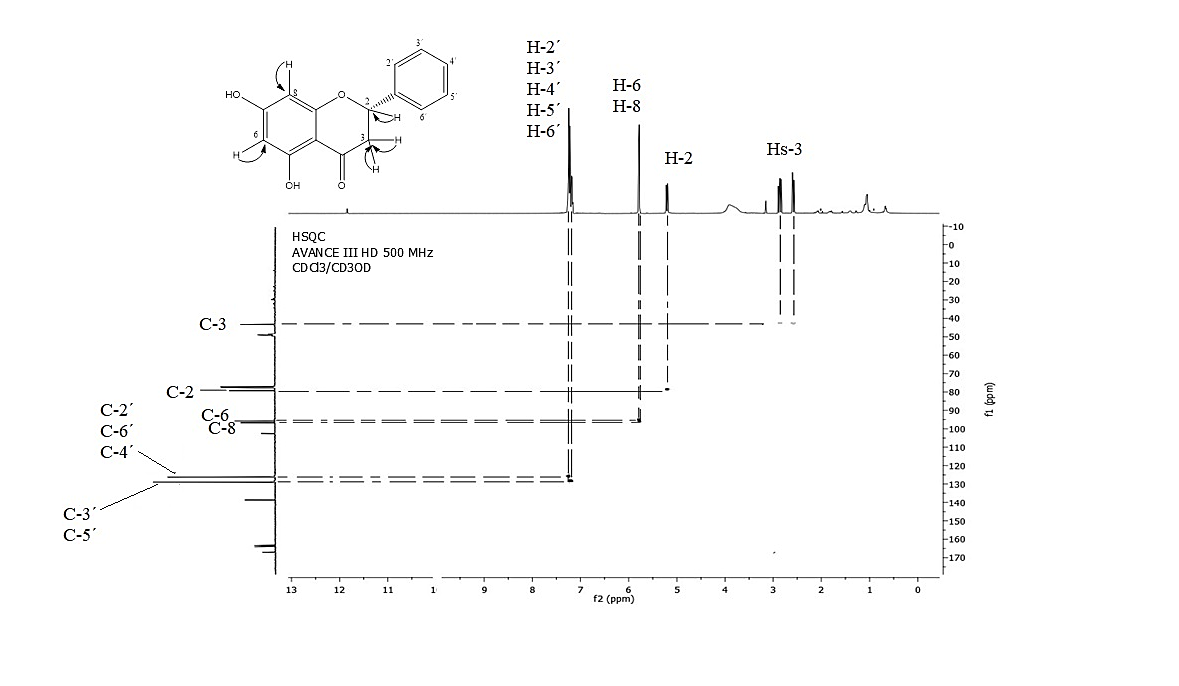


Figure S5. HSQC (500 MHz, CDCl_3_-CD_3_OD) of 5,7-dihydroxy flavanone (Pinocembrin, **1**).
